# Prognostic Value of the Circumferential Resection Margin in Esophageal Cancer Patients After Neoadjuvant Chemoradiotherapy

**DOI:** 10.1245/s10434-015-4827-2

**Published:** 2015-08-28

**Authors:** J. B. Hulshoff, Z. Faiz, A. Karrenbeld, G. Kats-Ugurlu, J. G. M. Burgerhof, J. K. Smit, J. Th. M. Plukker

**Affiliations:** Department of Surgical Oncology, University of Groningen, University Medical Center Groningen, 9713 AV Groningen, The Netherlands; Department of Pathology, University of Groningen, University Medical Center Groningen, 9713 AV Groningen, The Netherlands; Department of Epidemiology, University of Groningen, University Medical Center Groningen, 9713 AV Groningen, The Netherlands

## Abstract

**Background:**

Circumferential resection margins (CRM) for esophageal cancer (EC), defined by the College of American Pathologists (CAP; >0 mm) or the Royal College of Pathologists (RCP; >1 mm) as tumor-free (R0), are based on a surgery-alone approach. We evaluated the usefulness of both definitions in current practice with neoadjuvant chemoradiotherapy (nCRT).

**Methods:**

CRMs were measured in 209 patients (104 with nCRT) with locally advanced EC after transthoracic esophagectomy. Local recurrence and cancer related death were scored as events. Patients were followed for at least 2 years or until death. Prognostic factors (*P* < 0.1 in univariate analyses) for 2-year disease-free survival (DFS) and local recurrence-free survival (LRFS) were incorporated in multivariate Cox regression analyses. Both CRM measurements were analyzed separately and prognostic cutoff values (0–1.0 mm) were assessed in both groups.

**Results:**

Independent prognostic factors (*P* < 0.05) for 2-year DFS were tumor length, lymph node ratio, angioinvasion, and CAP R0 in the surgery-alone group and pN stage (*P* < 0.01) in the nCRT group. Prognostic factors (*P* < 0.05) for 2-year LRFS were CAP, lymph node ratio, and tumor length in the surgery-alone group, and CAP and grade in the nCRT group. Optimal CRM cutoff values between 0.0 and 0.2 mm were prognostic for 2-year DFS in the surgery-alone and at 0.3 mm for the nCRT group.

**Conclusions:**

nCRT affected the CRM cutoff values. After nCRT, the CRM R0 according to the CAP was only prognostic for 2-year LRFS. However, in the surgery-alone group, it was prognostic for both the 2-year DFS and LRFS.

Even with neoadjuvant chemoradiotherapy (nCRT), the overall 5-year survival rate after esophagectomy remains relatively low at 47 % in patients with locally advanced esophageal cancer (EC).^1^ A strong prognostic indicator after a curative intended esophagectomy is the circumferential resection margin (CRM), rendered as microscopic tumor-free (R0) or tumor-positive (R1).^2^–^8^ Commonly used definitions of a circumferentially R0 resection are those of the College of American Pathologist (CAP; CRM >0 mm) and the Royal College of Pathologists (RCP; CRM >1 mm).^9^,^10^ After nCRT, the optimal CRM may be influenced by tumor downsizing, which facilitates a R0 resection.^1^

The optimal CRM cutoff point after nCRT has not been defined yet. Recently, two meta-analyses showed a significant association of a positive CRM according to both definitions with poor outcome, which was even worse in patients with stage T3 disease or after nCRT.^11^,^12^ However, these studies did not assess which CRM definition was more powerful after nCRT, while contradictory results after nCRT were reported in three other studies without a surgery-alone control group.^13^–^15^ Two studies, with only squamous cell carcinoma, showed a significant better survival rate in patients with a CRM > 1 mm, whereas no survival benefit was observed in R0 resections according to the CAP and RCP in a study with only T3 stage adenocarcinomas.^13^–^15^

We assessed the optimal CRM cutoff point and the prognostic value of R0 resections according to the CAP and RCP criteria in EC patients treated either with nCRT or surgery alone.

## Patients and Methods

Data collection of this explorative retrospective study was provided from a prospective maintained database of EC patients according to the national guidelines and the rules approved by the local ethical commission (www.ccmo.nl). We included only patients with a locally advanced curatively resectable EC (stage II-III) treated between 1997 and 2013, in whom the CRM was adequately assessed by our expert pathologists. Of the patients treated with nCRT (*n* = 127) between 2005 and 2013, 23 were excluded because of the following criteria: incomplete medical records (*n* = 0), postoperative mortality (death within 90 days or in-hospital, *n* = 10), progressive disease within 3 months after surgery or microscopic irradical (R1; tumor cells <1 mm) longitudinal margins (*n* = 0) or follow-up <24 months (*n* = 13). Based on these exclusion criteria, a reference group of surgery-alone treated patients (*n* = 105) was constructed. Patients and tumor-related factors were matched and were equally distributed between both groups (Table 1).

**Table 1:** Patient characteristics in the surgery-alone and neoadjuvant chemoradiotherapy (nCRT) groups

	nCRT (*n* = 104)	Surgery alone (*n* = 105)	*P* value
Male	79 (76.0 %)	82 (78.1 %)	0.714^a^
Age (year), median (IQR)	63 (56–67)	64 (57–69)	0.228^b^
Histology			0.382^a^
Adenocarcinoma	88 (84.6 %)	84 (80 %)	
Squamous cell carcinoma	16 (15.4 %)	21 (20 %)	
Tumor location			0.654^a^
Middle esophagus	8 (7.7 %)	12 (11.4 %)	
Distal esophagus	50 (48.1 %)	49 (46.7 %)	
GEJ	46 (44.2 %)	44 (41.9 %)	
Tumor length >5 cm	59 (56.7 %)	60 (57.1 %)	0.695^b^
cT-stage			0.221^a^
T2	16 (15.4 %)	9 (8.6 %)	
T3	83 (79.8 %)	93 (88.6 %)	
T4a	5 (4.8 %)	3 (2.9 %)	
cN-stage			0.176^a^
N0	27 (26 %)	41 (39 %)	
N1	50 (48.1 %)	44 (41.9 %)	
N2	22 (21.2 %)	18 (17.1 %)	
N3	5 (4.8 %)	2 (1.9 %)	
pT-stage			<0.001^c^
Tx	1 (1 %)		
T0	21 (20.2 %)		
T1	22 (21.2 %)		
T2	14 (13.5 %)	20 (19 %)	
T3	46 (44.2 %)	82 (78.1 %)	
T4a	0 (0 %)	3 (2.9 %)	
pN-stage			<0.001^a^
N0	62 (59.6 %)	28 (26.7 %)	
N1	26 (25.0 %)	34 (32.4 %)	
N2	11 (10.6 %)	25 (23.8 %)	
N3	5 (4.8 %)	18 (17.1 %)	
Perineural growth	22 (21.2 %)	33 (31.4 %)	0.084^a^
Angioinvasion	22 (21.2 %)	51 (48.6 %)	<0.001^a^
Number of LN (>4 LN+)	10 (9.6 %)	32 (30.5 %)	<0.001^a^
Lymph node ratio (>0.2)	18 (17.3 %)	50 (47.6 %)	<0.001^a^
Follow-up mo, median (IQR)	27.5 (15.0–42.0)	29 (15.5–56.0)	0.241^b^
Tumor recurrence	63 (60.6 %)	75 (71.4 %)	0.098^a^
Local recurrence	17 (16.3 %)	35 (33.3 %)	0.005^a^
Death	60 (57.7 %)	83 (79 %)	0.001^a^
Tumor-related death	54 (51.9 %)	73 (69.5 %)	0.009^a^
CRM (mm), median (IQR)	3.3 (1.0–5.0)	0.5 (0–1.4)	<0.001^b^
0	9 (8.7 %)	27 (25.7 %)	<0.001^a^
0–1	13 (12.5 %)	40 (38.1 %)	<0.001^a^
>1	82 (78.8 %)	38 (36.2 %)	<0.001^a^

*nCRT* neoadjuvant chemoradiotherapy, *cT* clinical T stage, *cN* clinical lymph node stage, *pT* pathological T stage, *pN* pathologic lymph node stage, *LN* lymph node, *CRM* circumferential resection margin, *CAP* College of American Pathologists, *RCP* Royal College of Pathologists, *IQR* interquartile range

^a^ *χ* ^2^ test

^b^Mann–Whitney test

^c^Fisher exact test

Tumors staged according to the 6th TNM edition were recoded into the 7th edition.^16^,^17^ Before 2000 (*n* = 11), staging consisted of endoscopic ultrasonography (EUS) with fine-needle aspiration (FNA), computed tomography (CT) of the neck, thorax, and abdomen and occasionally 18-F-fluorodeoxyglucose positron emission tomography (FDG-PET, *n* = 8). After 2000, a standard FDG-PET was added, which was replaced by FDG-PET/CT after 2009. Two weeks after nCRT, patients were restaged with a CT thorax and abdomen.

### Treatment

All patients underwent a transthoracic esophagectomy with en bloc dissection of regional mediastinal and abdominal (including the celiac trunk region) lymph nodes. Patients with nCRT were treated according to the Dutch Chemoradiotherapy for Oesophageal Cancer Followed by Surgery Study (CROSS) regimen, consisting of intravenous paclitaxel (50 mg/m^2^) and carboplatin (AUC: 2 ml/min), administered five times during a 5-week concurrent radiation period (41.4 Gy/23 fractions of 1.8 Gy).^1^ Before 2009, patients received nCRT based on their participation in the CROSS trial, from 2009 onwards nCRT became standard of care for locally advanced EC patients (T1-4aN1-3, T2-4aN0-3; *n* = 75).

### Pathology

Resected specimens were examined according to a standardized protocol by two specialized gastrointestinal pathologists. The resected specimen was pinned on a Styrofoam plate by the surgeon, enabling accurate pathological assessment of the marked Clinical Tumor Volume and Gross Tumor Volume areas in patients treated with nCRT.^18^ CRM was measured according to the method of Quirke; the specimens were inked with Indian ink and fixed in formalin during 24 h.^6^ The specimens were sliced into transverse cross-sections of 0.5 cm for macroscopic assessment and sampling of at least two sections with the smallest CRM.^2^ The CRM was microscopically assessed on hematoxylin and eosin stained samples in tenths of millimetres. Furthermore, the pT-stage, pN-stage, the lymph-node ratio (>0.2 metastatic lymph node ratio), number of positive lymph nodes (>4), histological tumor type, tumor grade, angioinvasion, and perineural tumor growth were assessed.

### Follow-up

Patients were followed for at least 2 years or until death, every 3 months during the first year after surgery, every 6 months in the second year, and every year thereafter for the next 10 years. Tumor recurrence was defined as histo/cytologically proven, suspected radiological imaging, or clinically evident recurrence. Local recurrence included recurrent disease at the anastomotic site or in the original tumor/mediastinal bed.

### Statistical Analysis

Distribution of continuous patient characteristics was reported as median [interquartile range] and categorical variables were reported in numbers and percentages. The patients groups were compared with the Mann–Whitney test for continuous variables and *χ*^2^ or Fisher exact test for categorical response variables. Kaplan–Meier curves and log-rank test determine the 5-year disease-free survival (DFS) and local recurrence-free survival (LRFS) of both CRM definitions. Prognostic values of all variables for 2-year DFS were assessed with univariate Cox regression analysis. Factors within the univariate analysis were: age, tumor type, and grade (G1–2 vs. G3–4), clinical T and N stage, tumor length (>5 cm, measured endoscopic or with CT), treatment type (nCRT or surgery alone), and pathologic outcome: T and N stage, number of LN metastases (>4), and metastatic lymph node ratio (>0.2), perineural growth, and angioinvasion. Multivariate Cox regression was performed by incorporating all variables with a *P* value <0.1 on univariate analysis. Both, the CAP (CRM >0 mm) and RCP (CRM >1 mm) definition entered the multivariate analysis separately. The prognostic value of R0 resections according to the RCP and CAP for the 2-year DFS and 2-year LRFS was assessed with multivariate Cox regression analyses in both treatment groups. To assess the optimal cutoff value of the CRM on 2-year DFS, an explorative analysis was performed in both groups. Univariate analyses were undertaken to assess the prognostic value of all cutoff values (from 0.0 to 1.0 mm). The observed interval is based on the assumption that the expected optimal CRM cutoff should be between 0.0 and 1.0 mm. The Akaike Information Criterion (AIC), which quantifies the quality of a statistical model for a set of data was used to indirectly compare the prognostic value of the CAP and RCP model.^19^ It penalizes the number of explanatory variables by adding twice the number of variables in the model to the −2 log likelihood; in a formula AIC = −2 log likelihood +2 k, in which k is the number of explanatory variables in the model. The model with the lowest AIC was considered to be most prognostic. The backwards likelihood ratio method was used in the Cox regression analysis. Analyses were performed with SPSS version 22.

## Results

Patient characteristics are summarized in Table 1. All nCRT patients with CAP-R1 resections (*n* = 9; 8.7 %) had stage pT3. Of the 27 (25.7 %) R1 resections in patients treated with surgery alone, 24 had stage pT3, 1 had pT2, and 2 had stage pT4a disease. The median CRM differed significantly with 3.3 [interquartile range (IQR) 1.0–5.0] mm versus 0.5 (IQR 0–1.4) mm for the nCRT and surgery-alone group, respectively. The median follow up was 29.0 (IQR 15.5–56.0) months and 27.5 (IQR 15.0–42.0) in the surgery-alone and nCRT groups, respectively.

### Prognostic Value of the CAP and RCP Criteria

**Fig. 1:**
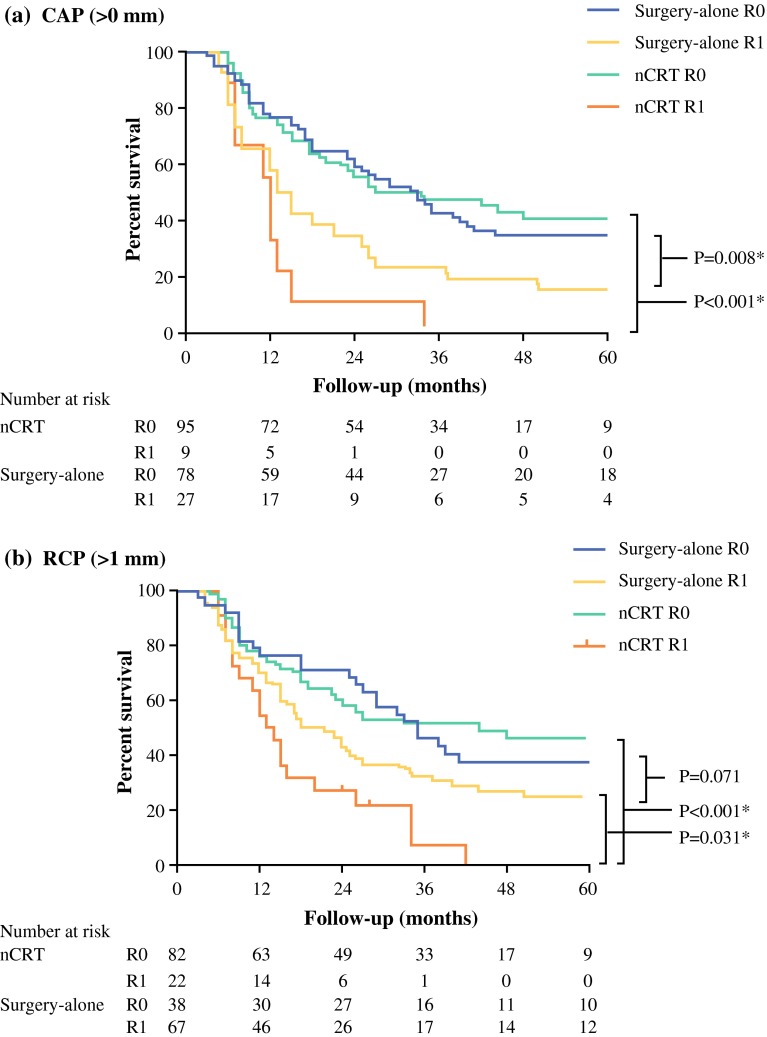
Disease-free survival in patients treated with neoadjuvant chemoradiotherapy and surgery-alone, with circumferential microscopic tumor-free (R0) or involved resection margins (R1), according to **a** CAP (0 mm) and **b** RCP (1 mm)

Figure 1 displays the DFS of both treatment groups, with a R0 resection or involved CRM (R1 resection) according to CAP (Fig. 1a) and RCP (Fig. 1b). With the log-rank test, the CAP definition was prognostic for 5-year DFS in both the surgery (*P* = 0.008) and nCRT group (*P* < 0.001) and the RCP definition was prognostic in the nCRT group (*P* < 0.001) but not in the surgery group (*P* = 0.071). The 5-year DFS was not different (*P* = 0.131) between CAP R1 patients treated with or without nCRT but differed (*P* = 0.031) between patients with an RCP R1 resection in both groups.

Table 2 displays all prognostic factors with a *P* < 0.1 on univariate analysis and Table 3 shows the multivariate Cox regression models containing either the CAP or RCP for 2-year DFS and LRFS in both groups. Independent prognostic factors for 2-year DFS in the surgery-alone group were tumor length [*P* = 0.006, hazard ratio (HR) 2.68, CI 1.33–5.43], lymph node ratio (*P* = 0.047, HR 2.57, CI 1.01–6.51), and CAP (*P* = 0.012, HR 0.41, CI 0.21–0.83). Independent prognostic factors for 2-year LRFS were lymph node ratio (*P* = 0.020, HR 3.11, CI 1.20–8.09), tumor length (*P* = 0.002, HR 10.99, CI 2.49–48.43), and CAP (*P* = 0.004, HR 0.27, CI 0.11–0.658). Both for 2-year DFS and LRFS, the model containing the CAP had a lower AIC than the RCP model and therefore was more prognostic.

**Table 2:** Prognostic factors with *P* < 0.1 on univariate analysis for disease-free and local recurrence-free survival in the surgery-alone and neoadjuvant chemoradiotherapy groups

Surgery-alone group						
	2-year DFS			2-year LRFS		
	HR	95 % CI	*P* value	HR	95 % CI	*P* value
pN0	1.00		0.001^a^	1.00		0.007^a^
pN1	3.36	1.10–10.32	0.034	4.08	0.87–19.26	0.076
pN2	7.36	2.47–21.92	0.000	3.39	0.62–18.56	0.160
pN3	8.05	2.58–25.05	0.000	11.54	2.48–53.79	0.002
Tumor length	2.23	1.14–4.34	0.019	8.45	1.97–36.21	0.004
Perineural growth	2.00	1.11–3.60	0.021			NS
Angioinvasion	2.90	1.49–5.65	0.002			NS
Number of LN	4.01	2.21–7.26	<0.001	3.41	1.46–7.97	0.005
Lymph node ratio	3.96	2.08–7.55	<0.001	3.51	1.44–8.57	0.006
CAP R0	0.45	0.25–0.82	0.010	0.42	0.18–0.98	0.044
RCP R0	0.46	0.23–0.91	0.025	0.52	0.20–1.32	0.168

nCRT group						
	2-year DFS			2-year LRFS		
	HR	95 % CI	*P* value	HR	95 % CI	*P* value
cT2	1.00		0.013^a^			NS
cT3	5.13	1.24–21.22	0.024			
cT4a	12.98	2.36–71.45	0.003			
pT0	1.00		0.054^a^			NS
pT1	1.13	0.36–3.49	0.837			
pT2	1.83	0.61–5.44	0.279			
pT3	2.74	1.13–6.64	0.025			
pN0	1.00		<0.001^a^	1.00		0.047^a^
pN1	2.58	1.30–5.13	0.007	0.56	0.07–4.65	0.590
pN2	4.69	2.14–10.30	0.000	5.37	1.28–22.55	0.022
pN3	6.87	2.47–19.11	0.000	7.31	0.76–70.88	0.086
Perineural growth	1.87	0.97–3.38	0.062			NS
Angioinvasion	1.81	0.98–3.53	0.055			NS
Number of LN	3.90	1.90–7.98	<0.001	8.00	1.92–33.25	0.004
Lymph node ratio	2.78	1.48–5.20	0.001	4.31	1.25–14.95	0.021
CAP R0	0.28	0.13–0.61	0.001	0.42	0.18–0.98	<0.001
RCP R0	0.40	0.22–0.74	0.003	0.30	0.09–1.06	0.061

*DFS* disease-free survival, *LRFS* local recurrence free survival, *CI* confidence interval, *cT* clinical T stage, *cN* clinical lymph node stage, *pT* pathological T stage, *pN* pathologic lymph node stage, *LN* lymph node, *CRM* circumferential resection margin, *R0* tumor-free resection margin, *CAP* College of American Pathologists, *RCP* Royal College of Pathologists, *NS* not significant

^a^Overall *P* value of the categorical variables

**Table 3:** Multivariate analysis of models containing the CRM definition according to the CAP (CRM 0 mm) or the RCP (CRM 1 mm), in the surgery-alone and neoadjuvant chemoradiotherapy groups

Surgery-alone group							
	2-year DFS				2-year LRFS		
	HR	95 % CI	*P* value		HR	95 % CI	*P* value
CAP model (AIC = 317.0)				CAP model (AIC = 168.2)			
CAP	0.41	0.21–0.83	0.012^a^	CAP	0.27	0.11–0.658	0.004^a^
LN ratio	2.57	1.01–6.51	0.047^a^	LN ratio	3.11	1.20–8.09	0.020^a^
Tumor length	2.68	1.33–5.43	0.006^a^	Tumor length	10.99	2.49–48.43	0.002^a^
Angioinvasion	1.90	0.94–3.85	0.075				
No. of LN+	2.13	0.92–4.95	0.078				
RCP model (AIC = 320.9)				RCP model (AIC = 174.2)			
RCP	0.83	0.38–1.78	0.627	RCP	0.57	0.22–1.51	0.258
LN ratio	2.68	1.05–6.81	0.039^a^	LN ratio	3.13	1.12–8.24	0.021^a^
Angioinvasion	1.95	0.94–4.03	0.072				
Perineural growth	1.89	0.99–3.59	0.053				
Tumor length	2.52	1.25–5.09	0.010^a^	Tumor length	8.59	1.99–37.08	0.004^a^
No. of LN	1.92	0.84–4.39	0.123				

nCRT group							
	2-year DFS				2-year LR		
	HR	95 % CI	*P* value		HR	95 % CI	*P* value
CAP model (AIC = 349.9)				CAP model (AIC = 73.5)			
CAP	0.47	0.18–1.23	0.124	CAP	0.06	0.01–0.31	0.001^a^
cT	3.20	0.76–13.49	0.114				
pN0	1.00		0.004^a,b^	Grade	16.91	2.12–135.05	0.008^a^
pN1	2.70	1.31–5.59	0.007				
pN2-3	3.39	1.43–8.03	0.005				
RCP model (AIC = 350.3)				RCP model (AIC = 80.0)			
RCP	0.69	0.31–1.52	0.359	RCP	1.01	0.08–13.50	0.995
cT	1.00		0.275	pN0–1	1.00		0.203
				pN1–2	8.81	0.31–252.23	
	2.32	0.51–10.51					
pN0	1.00		0.014^a,b^	Grade	30.07	2.79–324.60	0.005^a^
pN1	2.51	1.22–5.21	0.014				
				No. of LN	0.73	0.06–8.94	0.804
pN2–3	2.99	1.23–7.32	0.016				
pT0–1	1.00		0.359^b^	LN ratio	2.60	0.44–15.42	0.294
pT2	1.96	0.70–5.49	0.202				
pT3–4a	1.84	0.71–4.73	0.209				

*DFS* disease-free survival, *LRFS* local recurrence-free survival, *CI* confidence interval, *SCC* squamous cell carcinoma, *cT* clinical T stage, *cN* clinical lymph node stage, *pT* pathological T stage, *pN* pathologic lymph node stage, *LN* lymph node, *CRM* circumferential margin, *R0* tumor-free resection margin, *CAP* College of American Pathologists, *RCP* Royal College of Pathologists

^a^Significant (*P* < 0.05)

^b^Overall *P* value of the categorical variables

The only independent prognostic factors for 2-year DFS in the nCRT group was the pN-stage (overall *P* = 0.004), pN1 (*P* = 0.007, HR 2.70, CI 1.31–5.59), and pN2–3 (*P* = 0.005, HR 3.39, CI 1.43–8.03). Both CAP (*P* = 0.001, HR 0.06, CI 0.01–0.31) and tumor grade (*P* = 0.008, HR 16.91, CI 2.12–135.05) were prognostic for 2-year LRFS. For both 2-year DFS and LRFS, the multivariate regression model containing the CAP definition had a lower AIC and therefore was more prognostic.

### Optimal CRM after Surgery Alone and after nCRT

CRM cutoff values of 0.0 (*P* = 0.012, HR = 0.41, CI 0.21–0.83, AIC = 317.0), 0.1 (*P* = 0.045, HR = 0.50, CI 0.25–0.98, AIC = 320.0), and 0.2 mm (*P* = 0.028, HR = 0.48, CI 0.25–0.92, AIC = 318.8) were independent prognostic factors for 2-year DFS in the surgery-alone group. Based on the AIC, the 0.0-mm cutoff value (CAP) was the most prognostic. However, in the nCRT group, the optimal cutoff value for 2-year DFS was 0.3 mm (*P* = 0.045, HR = 0.35, CI 0.13–0.98, AIC = 348.1).

## Discussion

The prognostic value of the circumferential margin (CRM) has been proven in EC patients after surgery alone, but its significance after neoadjuvant treatment is not well defined yet. This study conducted in stage II-III EC patients showed that both definitions of a free CRM were not prognostic for 2-year DFS in patients treated with nCRT. The CAP definition (>0 mm), however, was an independent prognostic factor for 2-year DFS in the surgery-alone group and for LRFS in the nCRT and surgery-alone group. The optimal CRM cutoff value for 2-year DFS was 0.3 and between 0.0 and 0.2 mm in the nCRT and surgery-alone group, respectively.

This study is one of the first to assess the optimal cutoff value of the CRM after nCRT; previously published studies used either the RCP or CAP criteria of a free CRM. Although neoadjuvant treatment decreases the rate of R1 resection by transversal and sagittal tumor reduction, the induced fibrosis may contain different amounts of undetectable viable tumor cells.^1^ Therefore, the CRM assessment depends upon accurate histological examination of residual tumor, which might be related to tumor heterogeneity. CRM >1 mm showed to be prognostic, but several studies reported conflicting results in patients treated with nCRT (Table 4). Chao et al. described a significantly better disease-free and disease-specific survival, whereas Liu et al. noted a significantly better overall survival (OS).^13^,^14^ However, Harvin et al. failed to prove a survival benefit after nCRT with respect to both CAP and RCP–CRM resections.^15^ This difference might be explained by the inclusion of different pathologic tumor types; Harvin et al. only included ypT3 or higher adenocarcinomas, whereas Chao et al. and Liu et al. included only patients with squamous cell carcinomas.^13^–^15^ In our study, histologic tumor type did not to affect the prognostic value of the CRMs for DFS and LRFS, although the number of squamous cell carcinomas in the nCRT group was rather small (*n* = 16). Inclusion of pathologic T3 tumors in determining the optimal CRM seems comprehensible as circumferential R1 resections in pT2 tumors are generally considered to be caused by inadequate surgery.^7^,^20^,^21^ Moreover, Rao et al. stated that CRM involvement in the EC specimen is related to advanced disease rather than being an indicator of completeness of resection.^4^ In our study, only one patient staged as ypT2 disease had a R1 resection, due to extensive angioinvasive tumor growth within the CRM, which depends more on biologic aggressiveness rather than poor surgery. Another factor that might influence the CRM is the used surgical method; Suttie et al. noted that the transhiatal approach resulted in significantly more CRM involvement compared with the transthoracic approach.^22^ Because the transthoracic approach is our standard method, we could disregard this potential confounding effect.

**Table 4:** Studies regarding prognostic value of the circumferential resection margin after neoadjuvant chemoradiotherapy

Study (year)	Histology	Stage	Patients (n)	nCRT (%)	Outcome	CRM definition	*P* value^a^
Thompson et al.^23^	AC, SCC	cT1–4	240	124 (52 %)	5-year survival	RCP	NS
Chao et al.^13^	SCC	ypT3	151	151 (100 %)	LRFS	RCP	<0.05
					DFS	RCP	<0.05
					DSS	RCP	<0.05
Harvin et al.^15^	AC	ypT3	160	160 (100 %)	OS, DFS, LRFS	CAP	NS
					OS, DFS, LRFS	RCP	NS
Reid et al.^25^	AC SCC	cT1–4	269	42 (16 %)	DFS	RCP	<0.01
					OS	RCP	0.05
O’Farrell et al.^24^	AC, SCC, others	cT3	157	82 (52 %)	OS	RCP	NS
					OS	CAP	0.02
Liu et al.^14^	SCC	cT1–4	94	94 (100 %)	OS	RCP	<0.01

*SCC* squamous cell carcinoma, *AC* adenocarcinoma, *nCRT* neoadjuvant chemoradiotherapy, *cT* clinical T stage, *ypT* pathologic T stage after nCRT, *DFS* disease-free survival, *LRFS* local recurrence-free survival, *DSS* disease-specific survival, *CRM* circumferential resection margin, *CAP* College of American Pathologists, *RCP* Royal College of Pathologists

^a^Multivariate analysis

Three other studies assessed the value of the CRM in which only a part of the included patients received nCRT, again with conflicting results.^23^–^25^ Thompson et al. (*n* = 240, 52 % nCRT) did not find a survival benefit, whereas Reid et al. (*n* = 269, 15,6 % nCRT) found a significantly better DFS and OS in patients with a RCP R0 resection.^23^,^25^ Farrell et al. (*n* = 157, 52 % nCRT) found the CAP definition (*P* = 0.02) more prognostic for the OS than the RCP definition.^24^

As in patients treated with nCRT, the optimal CRM definition in surgically treated patients also is unclear. Two recent meta-analyses showed that both CRM definitions were associated with a poor survival, although the CAP criteria differentiated higher-risk groups.^11^,^12^ Moreover Chan et al. found that the CAP definition, based on the hazard ratio and subgroup analysis, had a prognostic advantage over the RCP criteria.^12^ Concordant to these results, we found that the optimal CRM cutoff value in the surgery-alone group, analyzed with the Akaike Information Criterion, was the CAP.

Beside the CRM, lymph node metastasis associated variables were important prognostic factors in this study; lymph node ratio >0.2 was independent prognostic for both 2-year DFS and LRFS in the surgery-alone group and pN-stage was the only prognostic factor for 2-year DFS in the nCRT group. One meta-analysis, which underlined the importance of lymph node metastasis, indicated that nodal metastases appeared to negate the prognostic value of the CRM.^12^ Moreover, the presence of lymph node metastases and an involved CRM indicated a more advanced-staged disease.^26^ Another prognostic factor in surgery-alone patients was the tumor length, which is in correspondence with previously published data.^27^

Pultrum et al. assessed the optimal CRM in surgically treated patients using the area under the curve (AUC) analysis on receiver operating curves (ROC, which does not incorporate the time factor.^2^ A method that includes the time factor is the more complex time-dependent ROC method according to Heagerty et al.^28^ For our limited explorative study, however, we prefer to use multivariate Cox regression analysis and suggest validating the results in a larger cohort.

## Conclusions

This study showed that both definitions of a tumor-free CRM (CAP > 0 mm, RCP > 1 mm) were not prognostic for DFS in patients treated with nCRT. A free CRM according the CAP definition was prognostic for 2-year DFS in the surgery-alone group and an optimal CRM cutoff between 0.0 and 0.2 and at 0.3 mm in the surgery-alone and nCRT groups, respectively. These findings should be validated in a large, prospective study.
